# InSAR Signal and Data Processing

**DOI:** 10.3390/s20133801

**Published:** 2020-07-07

**Authors:** Mengdao Xing, Zhong Lu, Hanwen Yu

**Affiliations:** 1 National Laboratory of Radar Signal Processing, Xidian University, Xi’an 710071, China; 2 Roy M. Huffington Department of Earth Sciences, Southern Methodist University, Dallas, TX 75205, USA; zhonglu@mail.smu.edu; 3 Department of Civil and Environmental Engineering, University of Houston, Houston, TX 77004, USA; yuhanwenxd@gmail.com; 4 National Center for Airborne Laser Mapping, University of Houston, Houston, TX 77004, USA

## Abstract

We present here the recent advances in exploring new techniques related to interferometric synthetic aperture radar (InSAR) signal and data processing and applications.

## 1. Introduction

This Special Issue “InSAR Signal and Data Processing” of Sensors collects eleven articles from several InSAR researchers over several countries. The selected articles cover both InSAR signal processing techniques and their practical applications in Earth sciences. Readers of all levels will be able to gain a better understanding of InSAR as well as the when, the how, and the why of applying this technology.

## 2. Special Issue Contents

The first paper [1], “Polarimetric Stationarity Omnibus Test (PSOT) for Selecting Persistent Scatterer Candidates with Quad-Polarimetric SAR Datasets”, proposes the polarimetric stationarity omnibus test method for improving the spatial density and the phase quality of persistent scatterer (PS) points. The experimental results show that the proposed method can achieve the polarimetric optimization of the interferometric phase of the PS, suppress the sidelobe of the strong scatterer effectively, and hence better reveal the details of the ground object. The second article [2], “Coherent Markov Random Field-Based Unreliable DSM Areas Segmentation and Hierarchical Adaptive Surface Fitting for InSAR DEM Reconstruction”, proposes a novel InSAR digital elevation model reconstruction method using a digital surface model generated by an InSAR system with a coherent Markov random field technique. The comparison results shown in the experimental section indicate the superiority of the proposed algorithm. The third paper [3], “Multibaseline Interferometric Phase Denoising Based on Kurtosis in the NSST Domain”, and the fourth article [4], “Extended Phase Unwrapping Max-Flow/Min-Cut Algorithm for Multibaseline SAR Interferograms Using a Two-Stage Programming Approach”, focus on phase denoising and phase unwrapping techniques of multibaseline InSAR, respectively. The fifth paper [5], “Mining-Induced Time-Series Deformation Investigation Based on SBAS-InSAR Technique: A Case Study of Drilling Water Solution Rock Salt Mine”, shows an InSAR case study concerning salt extraction based on solution mining. The study applies the SBAS-InSAR technique to obtain the spatial–temporal characteristics of the ground subsidence caused by solution mining activities. The sixth paper [6], “Phase Difference Measurement of Under-Sampled Sinusoidal Signals for InSAR System Phase Error Calibration”, discusses the issue related to phase error calibration in spaceborne single-pass InSAR. The proposed method of the phase difference measurement of the high-frequency internal calibration signal of the InSAR system is suitable for the phase error calibration. As the highest elevation permafrost region in the world, the Qinghai-Tibet Plateau (QTP) permafrost is quickly degrading due to global warming, climate change, and human activities. The seventh article [7], “Permafrost Deformation Monitoring Along the Qinghai-Tibet Plateau Engineering Corridor Using InSAR Observations with Multi-Sensor SAR Datasets from 1997–2018”, presents an application using a time-series InSAR technique with multiple SAR datasets to monitor the permafrost ground deformation along the QTEP from 1997 to 2018. GaoFen-3 is a new Chinese InSAR remote sensing satellite. The eighth article [8], “ScanSAR Interferometry of the Gaofen-3 Satellite with Unsynchronized Repeat-Pass Images”, discusses interferometric analysis and processing methods for GaoFen-3 images in ScanSAR mode. The ninth paper [9], “A Highly Efficient Heterogeneous Processor for SAR Imaging”, concerns the hardware design of a SAR signal processor consisting of two 18×16 heterogeneous arrays that provide 115.2 GOPS throughput. In the tenth paper [10], “Monitoring the Land Subsidence Area in a Coastal Urban Area with InSAR and GNSS”, 34 scenes of Sentinel-1A SAR images are used for SBAS and PS processing to obtain the surface deformation field of a large region spanning the Shenzhen, China, and Hong Kong Special Administrative Regions. The last article [11], “Safe Helicopter Landing on Unprepared Terrain Using Onboard Interferometric Radar”, proposes an interferometric radar survey system for the generation of ground surface topography for helicopter landing sites. The system generates high-quality three-dimensional terrain surface topography data and estimates the slope of the site with the required accuracy.

## References

[1] Luo X., Wang C., Shen P. (2020). Polarimetric Stationarity Omnibus Test (PSOT) for Selecting Persistent Scatterer Candidates with Quad-Polarimetric SAR Datasets. Sensors.

[2] Qian Q., Wang B., Hu X., Xiang M. (2020). Coherent Markov Random Field-Based Unreliable DSM Areas Segmentation and Hierarchical Adaptive Surface Fitting for InSAR DEM Reconstruction. Sensors.

[3] Liu Y., Li S., Zhang H. (2020). Multibaseline Interferometric Phase Denoising Based on Kurtosis in the NSST Domain. Sensors.

[4] Zhou L., Lan Y., Xia Y., Gong S. (2020). Extended Phase Unwrapping Max-Flow/Min-Cut Algorithm for Multibaseline SAR Interferograms Using a Two-Stage Programming Approach. Sensors.

[5] Liu X., Xing X., Wen D., Chen L., Yuan Z., Liu B., Tan J. (2019). Mining-Induced Time-Series Deformation Investigation Based on SBAS-InSAR Technique: A Case Study of Drilling Water Solution Rock Salt Mine. Sensors.

[6] Yuan Z., Gu Y., Xing X., Chen L. (2019). Phase Difference Measurement of Under-Sampled Sinusoidal Signals for InSAR System Phase Error Calibration. Sensors.

[7] Zhang Z., Wang M., Wu Z., Liu X. (2019). Permafrost Deformation Monitoring Along the Qinghai-Tibet Plateau Engineering Corridor Using InSAR Observations with Multi-Sensor SAR Datasets from 1997–2018. Sensors.

[8] Sun Z., Yu A., Dong Z., Luo H. (2019). ScanSAR Interferometry of the Gaofen-3 Satellite with Unsynchronized Repeat-Pass Images. Sensors.

[9] Wang S., Zhang S., Huang X., An J., Chang L. (2019). A Highly Efficient Heterogeneous Processor for SAR Imaging. Sensors.

[10] Hu B., Chen J., Zhang X. (2019). Monitoring the Land Subsidence Area in a Coastal Urban Area with InSAR and GNSS. Sensors.

[11] Shimkin P.E., Baskakov A.I., Komarov A.A., Ka M.-H. (2020). Safe Helicopter Landing on Unprepared Terrain Using Onboard Interferometric Radar. Sensors.

